# The impact of metabolic overweight/obesity phenotypes on unplanned readmission risk in patients with COPD: a retrospective cohort study

**DOI:** 10.3389/fphys.2023.1290611

**Published:** 2023-11-28

**Authors:** Yang Tian, Luna Liu, Yuchen Li, Xiude Fan, Wanhong Wu, Yingzhou Shi, Jie Jiang, Zinuo Yuan, Hang Dong, Huijie Li, Qiuhui Xuan, Chao Xu

**Affiliations:** ^1^ Shandong Provincial Hospital, Shandong University, Jinan, Shandong, China; ^2^ Key Laboratory of Endocrine Glucose and Lipids Metabolism and Brain Aging, Department of Endocrinology, Shandong Provincial Hospital Affiliated to Shandong First Medical University, Ministry of Education, Jinan, Shandong, China; ^3^ Shandong Clinical Research Center of Diabetes and Metabolic Diseases, Jinan, Shandong, China; ^4^ Shandong Institute of Endocrine and Metabolic Diseases, Jinan, Shandong, China; ^5^ Shandong Engineering Laboratory of Prevention and Control for Endocrine and Metabolic Diseases, Jinan, Shandong, China; ^6^ Department of Statistics and Medical Records Management, Shandong Provincial Hospital Affiliated to Shandong First Medical University, Jinan, Shandong, China

**Keywords:** COPD, obesity, metabolic abnormality, aging, readmission

## Abstract

**Background:** There is an inconsistent association between overweight/obesity and chronic obstructive pulmonary disease (COPD). Considering that different metabolic characteristics exist among individuals in the same body mass index (BMI) category, the classification of overweight/obesity based on metabolic status may facilitate the risk assessment of COPD. Our study aimed to explore the relationship between metabolic overweight/obesity phenotypes and unplanned readmission in patients with COPD.

**Methods:** We conducted a retrospective cohort study using the Nationwide Readmissions Database (NRD). According to metabolic overweight/obesity phenotypes, patients were classified into four groups: metabolically healthy non-overweight/obesity (MHNO), metabolically unhealthy non-overweight/obesity (MUNO), metabolically healthy with overweight/obesity (MHO), and metabolically unhealthy with overweight/obesity (MUO). The primary outcome was unplanned readmission to hospital within 30 days of discharge from index hospitalization. Secondary outcomes included in-hospital mortality, length of stay (LOS) and total charges of readmission within 30 days.

**Results:** Among 1,445,890 patients admitted with COPD, 167,156 individuals were unplanned readmitted within 30 days. Patients with the phenotype MUNO [hazard ratio (HR), 1.049; 95%CI, 1.038–1.061; *p* < 0.001] and MUO (HR, 1.061; 95%CI, 1.045–1.077; *p* < 0.001) had a higher readmission risk compared with patients with MHNO. But in elders (≥65yr), MHO also had a higher readmission risk (HR, 1.032; 95%CI, 1.002–1.063; *p* = 0.039). Besides, the readmission risk of COPD patients with hyperglycemia or hypertension regardless of overweight/obesity increased (*p* < 0.001).

**Conclusion:** In patients with COPD, overweight/obesity alone had little effect on unplanned readmission, whereas metabolic abnormalities regardless of overweight/obesity were associated with an increased risk of unplanned readmission. Among the metabolic abnormalities, particular attention should be paid to hyperglycemia and hypertension. But in elders (≥65yr) overweight/obesity and metabolic abnormalities independently exacerbated the adverse outcomes.

## Introduction

COPD is the most prevalent chronic respiratory disease, resulting in the highest number of deaths related to chronic respiratory diseases globally and contributing significantly to the worldwide burden of disability-adjusted life years (López-Campos et al., 2016; Labaki and Han, 2020; Viegi et al., 2020). In the United States, approximately 700,000 patients with COPD are hospitalized every year, and almost 20% of them are readmitted within 30 days of discharge (Jencks et al., 2009; HCUPnet, 2022). Hospital readmission can have a negative impact on patients and the hospitals and is costly for both public and private payers (Fingar and Washington, 2006). Furthermore, it appears that 30-day readmission is associated with increased morbidity, possible mortality, and decline in quality of life (Vashi et al., 2013). Consequently, readmission within 30 days represents a crucial issue that warrants focused attention. In 2014, COPD was added to the Hospital Readmission Reduction Program by the Centers for Medicare and Medicaid Services (CMS), prompting hospitals to reduce 30-day readmissions of patients with COPD (Fingar and Washington, 2006). Therefore, identifying controllable factors to prevent unplanned readmission in COPD is urgent and necessary to alleviate the healthcare burden.

Over the past 50 years, obesity has reached epidemic levels and has increased the risk of many diseases, such as cardiovascular disease, stroke, cancers and so on (Blüher, 2019). In addition, obesity has been linked to respiratory conditions including obstructive sleep apnea, asthma, and pulmonary embolism (Zammit et al., 2010). However, the association between obesity and COPD still remains controversial. Some studies have reported that obesity is associated with a worse disease course in patients with COPD (Lambert et al., 2017), while several others suggested that obesity has neither a beneficial nor detrimental effect on the risk of exacerbation in COPD (Cecere et al., 2011; Salhi et al., 2015). Additionally, some studies have identified a significant protective effect of obesity on all-cause mortality in both community-dwelling and hospitalized patients with COPD, indicating that the “obesity paradox” may exist in obstructive pulmonary diseases (Spelta et al., 2018). Obesity is often accompanied by metabolic disorders, such as hypertension, dyslipidemia, and hyperglycemia. Moreover, the metabolic status varies considerably among individuals within the same BMI category. Notably, a significant proportion of individuals with obesity do not exhibit metabolic abnormalities, a condition known as metabolic healthy obesity (MHO). Many studies have indicated that metabolic abnormalities in patients with COPD aggravate respiratory symptoms and lung dysfunction (Cebron Lipovec et al., 2016; Price et al., 2016). Therefore, it is essential to categorize overweight/obesity into different subtypes based on metabolic health status, to eliminate the mutual influence of overweight/obesity and metabolic abnormalities.

To our knowledge, there is a lack of systematic studies on the association between metabolic overweight/obesity phenotypes and COPD. In this study, we utilized the NRD to investigate the impact of different metabolic overweight/obesity phenotypes on the risk of unplanned 30-day readmissions and readmission burden in COPD patients. In this way, controllable factors would be found and ultimately provide a reference for clinical prevention and intervention.

## Materials and methods

### Data source

We utilized the 2018 Nationwide Readmissions Database (NRD), a longitudinal and nationally representative database to conduct a retrospective cohort study. The NRD is an all-payer hospital inpatient stays database developed by the Agency for Healthcare Research and Quality for the Healthcare Cost and Utilization Project (HCUP). Based on HCUP State Inpatient Databases (SID), the NRD comprises reliable and verified patient linkage numbers that capable of tracking a patient between hospitals across the state within a year (NRD Overview, 2022). The 2018 NRD contains data from 28 states, representing 59.7 percent of the total U.S. resident population and encompassing 58.7 percent of all U.S. hospitalizations (NRD Overview, 2022). This study was considered exempt from Institutional Review Board since the NRD is a publicly available database that includes de-identified patient information, which was collected according to the data use agreement sponsored by AHRQ.

### Study population

**FIGURE 1:**
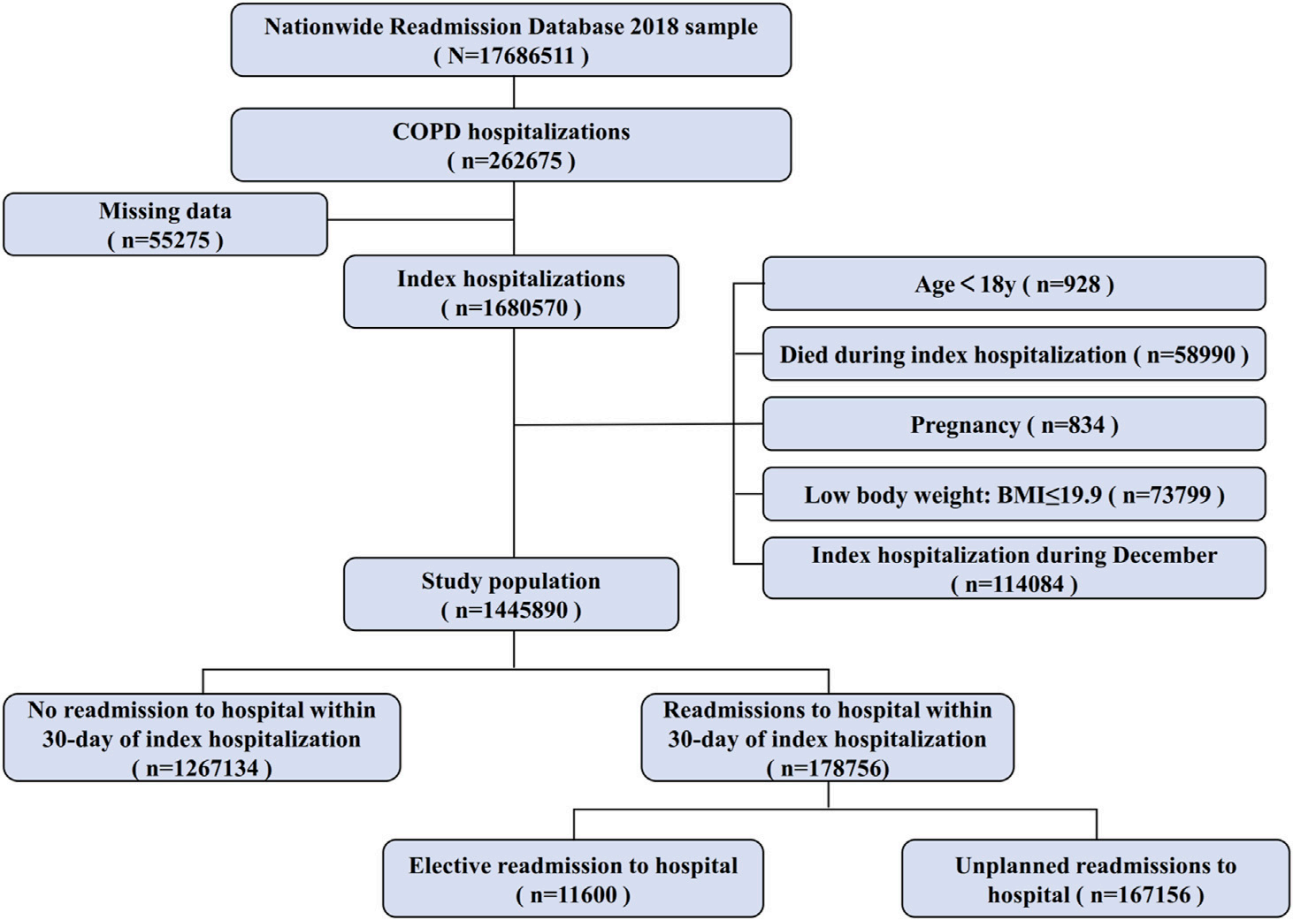
Flowchart of patient selection.

Our study contained all adults (age ≥ 18yr) hospitalized with the diagnosis of COPD, as defined by the International Classification of Diseases, Tenth Revision, Clinical Modification (ICD-10-CM) codes, between January-November 2018, at the time of index hospitalization. After the first admission with a discharge diagnosis of COPD, patients were deemed to be “at-risk” for hospitalization and contributed to follow-up time until 31 December 2018, or until death. We excluded patients who met the following criteria: (a) age < 18 at the time of index hospitalization, (b) index hospitalization during December 2018, (c) patients who died at the time of index hospitalization, (d) patients with low body weight (BMI ≤ 19.9 kg/m^2^) at the time of index hospitalization, (e) patients with pregnancy at the time of index hospitalization, (f) those with missing data for analyses. Finally, there were 428,637 unplanned 30-day readmissions following 1,445,890 index hospitalizations for COPD. The flowchart of patient selection from the NRD is shown in Figure 1.

### Data collection

Using NRD variables, we collected various patient characteristics, including age, sex, disposition of the patient, admission types (whether unplanned), emergency record, length of stay (LOS), primary expected payer (Medicare, Medicaid, private insurance, self-pay, no charge or others), patient location, resident (identifying the patient as a resident of the state in which they received hospital care), total charges, median household income, risk of mortality, severity of illness. Also, we collected data on respiratory intubation and mechanical ventilation from the Procedure Coding System (PCS). In addition, we calculated comorbidity score based on Charlson’s index of comorbidities, which assigns weights for various diseases (Charlson et al., 1987). The comorbidities included myocardial infarction, congestive heart failure, peripheral vascular disease, cerebrovascular disease, dementia, connective tissue disease, ulcer disease, mild liver disease, hemiplegia, moderate/severe renal disease, any tumor, leukemia, lymphoma, moderate/severe liver disease, metastatic solid tumor and Acquired Immune Deficiency Syndrome (AIDS). All the ICD-10 codes used above were shown in Supplementary Table S1.

### Exposure, outcomes, and definition

Body mass index (BMI) was classified according to World Health Organization criteria for adults, with overweight/obesity was defined as BMI ≥ 25 kg/m^2^. Metabolic status was defined based on the National Cholesterol Education Program Adult Treatment Panel III. Metabolically unhealthy patients had at least two of the following three components of metabolic syndrome: (Viegi et al., 2020): Hyperglycemia; (Labaki and Han, 2020); hyperlipidemia: high serum triglyceride levels or low high-density lipoprotein cholesterol levels, etc. and (López-Campos et al., 2016) hypertension. Waist circumference was excluded from the definition of metabolically unhealthy status due to the strong correlation between waist circumference and BMI. Individuals were categorized into four metabolic overweight/obesity phenotypes, based on their metabolic and overweight/obesity statuses: (Viegi et al., 2020): metabolically healthy non-overweight/obesity (MHNO): BMI < 25 kg/m^2^ and fewer than two metabolic syndrome components; (Labaki and Han, 2020); metabolically unhealthy non-overweight/obesity (MUNO): BMI < 25 kg/m^2^ and at least two metabolic syndrome components; (López-Campos et al., 2016); metabolically healthy overweight/obesity (MHO): BMI ≥ 25 kg/m^2^ and fewer than two metabolic syndrome components; and (HCUPnet, 2022) metabolically unhealthy overweight/obesity (MUO): BMI ≥ 25 kg/m^2^ and at least two metabolic syndrome components. Besides, individuals with COPD were identified according to ICD-10-CM diagnostic codes. The ICD-10-CM codes of these diagnoses are listed in Supplementary Table S1.

In this study, the primary outcome was unplanned all-cause readmission to hospital within 30 days of discharge from the index hospitalization. We defined hospital readmission as unplanned and all-cause readmission within 30 days of discharge from the index hospitalization, excluding planned or elective readmissions, such as those regular treatment. This definition was in accordance with the Hospital Readmission Reduction Program established by CMS, which integrated COPD into its framework in 2014, and followed the guidelines set by the HCUP. If a patient experienced multiple readmissions within 30 days, only the first was counted. Secondary outcomes of interest included in-hospital mortality, LOS, and total charges of readmission within 30 days.

### Statistical analysis

Descriptive statistics were employed to compare patient demographics and admission characteristics among the index hospitalization for different metabolic obesity phenotypes. We used one-way analysis or Kruskal–Wallis one-way analysis of variance testing to compare continuous variables and chi-square test to compare categorical variables. Continuous variables with a normal distribution were presented as the mean (standard deviation), while continuous variables with a skewed distribution were presented as the median (25th percentile, 75th percentile). Categorical variables were presented as numbers (percentages). To assess the independent effect of metabolic phenotypes on longitudinal outcomes, we conducted multivariable Cox proportional hazard analysis, adjusting for age, sex, disposition of patient, HCUP indicator of emergency department record, length of stay of the index hospitalization, expected primary payer, total charge of the index hospitalization, median household income for patient’s ZIP Codes, smoking, drinking, comorbidity score and respiratory intubation and mechanical ventilation. The selection of these covariates was based on both clinical relevance, supported by existing literature, and statistical significance, as determined by single-factor Cox regression analyses for each of the covariates (*p* < 0.05). Bonferroni correction was applied to all multiple comparisons in the study. A two-sided *p*-value < 0.05 was considered significant. SPSS 25.0 software (SPSS Inc., Chicago, IL, United States) was used to analyze the data.

## Results

### Baseline characteristics

From the 17,686,511 discharge records available in NRD 2018, a final sample of 1,445,890 COPD discharges was identified for our analysis. Table 1 provides a summary of the baseline characteristics of COPD patients with different metabolic overweight/obesity phenotypes during the index hospitalization. Among these patients, 541,684 patients (37.46%) were classified as MHNO, 90,940 (6.29%) were classified as MHO, 205,586 (42.03%) were classified as MUNO and 205,586 (14.22%) were classified as MUO.

**TABLE 1:** Patient- and hospitalization-characteristics of COPD patients with metabolic overweight/obesity phenotypes at time of index hospitalization.

	Non-obesity/overweight		Obesity/overweight		
Variables	MHNO	MUNO	MHO	MUO	*p*
No. of cases	541,684 (37.46)	607,680 (42.03)	90,940 (6.29)	205,586 (14.22)	
Age (years), median (IQR)	69 (59–78)a	73 (65–81)b	63 (55–72)c	67 (59–75)d	<0.001
18–44	20,777 (3.84) a	4,670 (0.77) b	6,414 (7.05) c	5,036 (2.45) d	
45–64	191,980 (35.44) a	140,645 (23.14) b	42,906 (47.18) c	78,592 (38.23) d	
≥65	328,927 (60.72) a	462,365 (76.09) b	41,620 (45.77) c	121,958 (59.32) d	
Female, n (%)	284,352 (52.49) a	295,421 (48.61) b	55,552 (61.09) c	114,482 (55.69) d	<0.001
Disposition of patient					<0.001
Routine, n (%)	314,974 (58.15) a	321,625 (52.93) b	52,150 (57.35) c	109,932 (53.47) d	
Transfer to Short-term Hospital, n (%)	5,781 (1.07) a	6,210 (1.02) a, b	947 (1.04) a, b	1991 (0.97) b	
Transfer Other, n (%)	105,565 (19.49) a	132,311 (21.77) b	17,570 (19.32) a	42,173 (20.51) b	
Home Healthcare, n (%)	103,322 (19.07) a	139,186 (22.90) b	18,719 (20.58) c	48,948 (23.81) d	
Against Medical Advice, n (%)	11,888 (2.19) a	8,204 (1.35) b	1,546 (1.70) c	2,523 (1.23) d	
Discharge alive, destination unknown, n (%)	154 (0.03) a	144 (0.02) a	8 (0.01) b	19 (0.01) b	
Emergency record, n (%)	421,708 (77.85) a	478,005 (78.66) b	69,683 (76.63) c	160,037 (77.84) a	<0.001
Length of stay of index admission (days), mean (SD)	5.44 (7.31) a	5.42 (6.42) a	6.12 (7.95) b	5.90 (6.84) c	<0.001
Primary expected payer					<0.001
Medicare, n (%)	366,849 (67.72) a	486,388 (80.04) b	54,989 (60.47) c	147,889 (71.94) d	
Medicaid, n (%)	71,052 (13.12) a	44,315 (7.29) b	15,235 (16.75) c	23,560 (11.46) d	
Private insurance, n (%)	70,076 (12.94) a	55,119 (9.07) b	15,083 (16.59) c	25,777 (12.54) d	
Self-pay, n (%)	16,936 (3.13) a	6,682 (1.10) b	2,814 (3.09) a	2,905 (1.41) b	
No charge, n (%)	2,505 (0.46) a	1,047 (0.17) b	452 (0.50) a	510 (0.25) b	
Other, n (%)	14,266 (2.63) a	14,129 (2.33) b	2,367 (2.60) a	4,945 (2.41) b	
Patient Location					<0.001
Large central counties, n (%)	123,237 (22.75) a	145,789 (23.99) b	20,529 (22.57) a	48,949 (23.81) b	
Large fringe counties, n (%)	127,407 (23.52) a	152,157 (25.04) b	22,300 (24.52) c	50,908 (24.76) b, c	
Medium metro counties, n (%)	124,951 (23.07) a	136,193 (22.41) b	20,793 (22.86) a	45,899 (22.33) b	
Small metro counties, n (%)	60,273 (11.13) a	66,242 (10.90) b	10,342 (11.37) a, c	23,857 (11.60) c	
Micropolitan counties, n (%)	58,387 (10.78) a	60,148 (9.90) b	10,100 (11.11) c	21,041 (10.23) d	
Not metro/micropolitan counties, n (%)	47,429 (8.76) a	47,151 (7.76) b	6,876 (7.56) b	14,932 (7.26) c	
Patient State is the same as Hospital State					
Nonresident, n (%)	26,927 (4.97) a	28,609 (4.71) b	4,549 (5.00) a	9,253 (4.50) b	<0.001
Resident, n (%)	514,757 (95.03) a	579,071 (95.29) b	86,391 (95.00) a	196,333 (95.20) b	
Total charges of index admission (dollars), mean (SD)	61,280.83 (100,701.86) a	65,739.76 (91,479.89) b	72,291.59 (112,241.03) c	72,484.60 (97,693.22) c	<0.001
Median household income					<0.001
0–25th percentile, n (%)	175,151 (32.33) a	190,826 (31.40) b	30,147 (33.15) c	68,034 (33.09) c	
26th to 5th percentile, n (%)	162,398 (29.98) a	179,850 (29.60) b	27,854 (30.63) c	63,262 (30.77) c	
51st to 75th percentile, n (%)	123,918 (22.88) a	140,348 (23.10) b	21,043 (23.14) a, b	46,977 (22.85) a, b	
76th to 100th percentile, n (%)	80,217 (14.81) a	96,656 (15.91) b	11,896 (13.08)c	27,313 (13.29) c	
Smoking, n (%)	197,720 (36.50) a	161,746 (26.62) b	29,085 (31.98) c	48,177 (23.43) d	<0.001
Drinking, n (%)	52,074 (9.61) a	29,109 (4.79) b	5,799 (6.38) c	6,809 (3.31) d	<0.001
Risk of mortality					<0.001
No class specified, n (%)	20 (0.00) a	21 (0.00) a	3 (0.00) a	3 (0.00) a	
Minor likelihood of dying, n (%)	141,956 (26.21) a	83,904 (13.81) b	23,681 (26.04) a	29,957 (14.57) b	
Moderate likelihood of dying, n (%)	158,690 (29.30) a	205,847 (33.87) b	24,963 (27.45) c	678,949 (33.02) d	
Major likelihood of dying, n (%)	171,858 (31.73) a	233,020 (38.35) b	29,708 (32.67) c	78,579 (38.22) b	
Extreme likelihood of dying, n (%)	69,160 (12.77) a	84,888 (13.97) b	12,585 (13.84) b	29,153 (14.18) b	
Severity of illness					<0.001
No class specified, n (%)	20 (0.00) a	21 (0.00) a	3 (0.00) a	3 (0.0) a	
Minor loss of function, n (%)	52,612 (9.71) a	32,998 (5.43) b	4,626 (5.09) c	5,803 (2.82) d	
Moderate loss of function, n (%)	200,481 (37.01) a	212,459 (34.96) b	28,787 (31.65) c	59,417 (28.90) d	
Major loss of function, n (%)	194,322 (35.87) a	254,381 (41.86) b	37,725 (41.48) b	97,212 (47.29) c	
Extreme loss of function, n (%)	94,249 (17.40) a	107,821 (17.74) b	19,799 (21.77) c	43,151 (20.99) d	
Comorbidity score, mean (SD)	1.83 (2.24) a	2.36 (2.11) b	1.72 (2.02) c	2.20 (1.88) d	<0.001
Comorbidity					
Myocardial infarction, n (%)	48,331 (8.92) a	114,375 (18.82) b	7,124 (7.83) c	32,901 (16.00) d	<0.001
Congestive heart failure, n (%)	122,370 (22.59) a	239,759 (39.45) b	29,780 (32.75) c	101,578 (49.41) d	<0.001
Peripheral vascular disease, n (%)	49,962 (9.22) a	87,877 (14.46) b	5,975 (6.57) c	18,772 (9.13) a	<0.001
Cerebrovascular disease, n (%)	30,395 (5.61) a	63,751 (10.49) b	3,560 (3.91) c	14,506 (7.06) d	<0.001
Dementia, n (%)	46,239 (8.54) a	63,501 (10.45) b	3,468 (3.81) c	9,328 (4.54) d	<0.001
Connective tissue disease, n (%)	24,226 (4.47) a	25,020 (4.12) b	4,548 (5.00) c	8,395 (4.08)b	<0.001
Ulcer disease, n (%)	10,772 (1.99) a	10,993 (1.81) b	1,434 (1.58) c	2,895 (1.41) d	<0.001
Mild liver disease, n (%)	45,107 (8.33) a	35,106 (5.78) b	8,327 (9.16) c	13,644 (6.64) d	<0.001
Hemiplegia, n (%)	9,886 (1.83) a	12,930 (2.13) b	1,393 (1.53) c	3,232 (1.57) b	<0.001
Moderate or severe renal disease, n (%)	131,985 (24.37) a	249,308 (41.03) b	24,702 (27.16) c	90,609 (44.07) d	<0.001
Any tumor, n (%)	70,540 (13.02) a	64,889 (10.68) b	8,709 (9.58) c	15,698 (7.64) d	<0.001
Leukemia, n (%)	4,270 (0.79) a	4,340 (0.71) b	547 (0.60) c	1,040 (0.51) d	<0.001
Lymphoma, n (%)	3,600 (0.66) a	3,050 (0.46) b	415 (0.50) b	754 (0.37) c	<0.001
Moderate or severe liver disease, n (%)	6,899 (1.27) a	4,622 (0.76) b	1,415 (1.56) c	1,532 (0.75) b	<0.001
Metastatic solid tumor, n (%)	22,864 (4.22) a	17,762 (2.92) b	2,459 (2.70) c	3,558 (1.73) d	<0.001
AIDS, n (%)	2,726 (0.50) a	1,201 (0.20) b	246 (0.27) c	297 (0.14) d	<0.001
Respiratory intubation and mechanical ventilation, n (%)	47,224 (8.72) a	50,406 (8.29) b	14,090 (15.49) c	30,740 (14.95) d	<0.001

Notes: The small letters (a, b, c, d) in this table refer to comparisons between groups. There is no statistical difference between groups with the same small letters. AIDS, acquired immune deficiency syndrome.

The median age of the study population was 70 years, and the MUNO population was the oldest among the four phenotypes. Regardless of the phenotype, the age group over 65 years old accounted for the largest proportion. Women accounted for 749,807 (51.9%) of the total study population. Both the LOS and hospitalization costs at index hospitalizations were higher in the MHO and MUO phenotypes compared to the other two phenotypes (*p* < 0.001). Among the four phenotypes, the MUNO group had the highest comorbidity score, followed by the MUO group. In addition, patients with MUNO had higher emergency department records than the other three groups (*p* < 0.001), and patients with obesity (MHO and MUO) were more likely to undergo respiratory intubation and mechanical ventilation (*p* < 0.001).

### The outcomes based on metabolic overweight/obesity phenotypes

**FIGURE 2:**
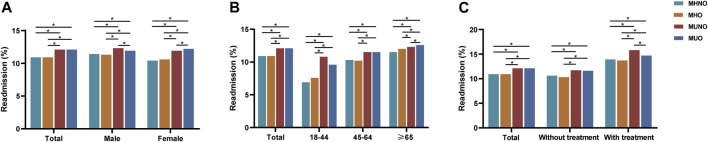
Readmission rates in patients with COPD with metabolic overweight/obesity phenotypes within 30 days. **(A)** In total population and by gender. **(B)** By age. **(C)** By treatment (respiratory intubation and mechanical ventilation). *The *p*-value of the inter-group comparison was less than 0.050. MHNO, metabolically healthy non-overweight/obesity; MUNO, metabolically unhealthy non-overweight/obesity; MHO, metabolically healthy overweight/obesity; MUO, metabolically unhealthy overweight/obesity.

In this study, we observed that the 30-day unplanned readmission rates in patients with MUNO (12.08%) and MUO (12.08%) were higher than those in MHO (10.85%) and MHNO (10.90%) (*p* < 0.001). However, results varied by gender, with the highest 30-day unplanned readmission rate for MUNO (12.30%) in males and the highest for MUO (12.24%) in females, although the 30-day unplanned readmission rate was higher in the metabolic abnormality patients than in the metabolically healthy patients in both males (MHNO, 11.44%; MUNO, 12.30%; MHO, 11.29%; MUO, 11.87%; *p* < 0.001) and females (MHNO, 10.40%; MUNO, 11.85%; MHO, 10.58%; MUO, 12.24%; *p* < 0.001) (Figure 2A). The results also varied by age group. The rate in the 18–44 age group (MHNO, 6.92%; MUNO, 10.77%; MHO, 10.23%; MUO, 9.57%; *p* < 0.001) and the 45–64 age group (MHNO, 10.32%; MUNO, 11.49%; MHO, 7.59%; MUO, 11.48%; *p* < 0.001) was consistent with the total study population, while the ≥ 65 age group (MHNO, 11.48%; MUNO, 12.28%; MHO, 12.00%; MUO, 12.57%; *p* < 0.001) showed a higher readmission rate in patients with obesity than in non-obese individuals for the same metabolic abnormalities (Figure 2B). In addition, 30-day unplanned readmission rate was higher in MUNO (15.80%) than in the other three phenotypes (MHNO, 13.91%; MHO, 13.70%; MUO, 14.72%) among patients with respiratory intubation and mechanical ventilation (Figure 2C).

There was no significant difference in in-hospital mortality within 30 days of readmission between metabolically healthy and unhealthy patients (Table 2). However, patients with overweight/obesity had lower in-hospital mortality than patients without overweight/obesity (Table 2). Concerning the length of stay for readmission within 30 days, the MHO group was the highest, while the other three groups had no significant difference. Regarding total charges for readmission within 30 days, patients with MHO, MUNO and MUO cost more than patients with MHNO (Table 2).

**TABLE 2:** Analysis of the relationships between metabolic obesity/overweight phenotypes and medical burden and costs within 30 days of discharge.

	Non-obesity/overweight		Obesity/overweight		
Secondary outcomes	MHNO	MUNO	MHO	MUO	*p*
Inpatient mortality	2.30%a	2.30%c	1.80%b	1.60%b	<0.01
Length of stay	2.39 (5.23)a	2.40 (5.22)a	2.52 (6.03)b	2.40 ((5.40)a	<0.01
Total charges	24,732.38 (66,246.20)a	26,301.16 (70,348.94)b	26,913.56 (81,046.04)b	26,567.67 (74,241.06)b	<0.01

Notes: The small letters (a, b, c, d) in this table refer to comparisons between groups. There is no statistical difference between groups with the same small letters.

### Cox regression analysis evaluating 30-day unplanned readmission risk

**FIGURE 3:**
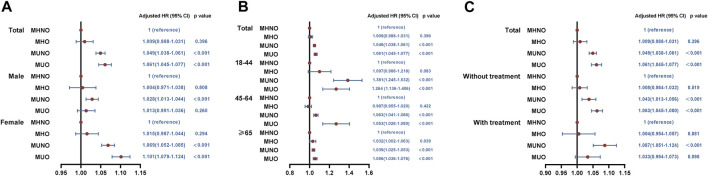
Results of cox regression analysis for the relationship between metabolic obesity phenotypes and 30-day readmissions. **(A)** In total population and by gender. **(B)** By age. **(C)** By treatment (respiratory intubation and mechanical ventilation). MHNO, metabolically healthy non-overweight/obesity; MUNO, metabolically unhealthy non-overweight/obesity; MHO, metabolically healthy overweight/obesity; MUO, metabolically unhealthy overweight/obesity.

In the total study population and subgroups, we investigated whether metabolic overweight/obesity phenotypes increased 30-day unplanned readmission risk (Figure 3), adjusting for covariates including age, sex, disposition of patient, emergency record, LOS, primary expected payer, patient location, resident, total charges, median household income, smoking, drinking, comorbidity score and respiratory intubation and mechanical ventilation. Compared to patients with MHNO, the readmission risk for patients with MUNO and MUO increased to 1.049 (95%CI, 1.038–1.061; *p* < 0.001) and 1.061 (95%CI, 1.045–1.077; *p* < 0.001) respectively (Figure 3). No difference was found in patients with MHO. Subgroup analyses were also conducted based on gender, age, and treatment. In the age ≥ 65 years group, the unplanned readmission risk was higher in either MHO (HR, 1.032; 95% CI, 1.002–1.063; *p* = 0.039), MUNO (HR, 1.039, 95% CI, 1.025–1.053; *p* < 0.001) or MUO (HR, 1.056; 95% CI, 1.036–1.076; *p* < 0.001) phenotype than in MHNO (Figure 3B). In addition, in patients with respiratory intubation and mechanical ventilation, only MUNO phenotype (HR, 1.087; 95%CI, 1.051–1.124; *p* < 0.001) had a higher unplanned readmission risk compared to the MHNO phenotype (Figure 3C). Other subgroups were consistent with the total study population.

### Further analysis of 30-day unplanned readmission

**FIGURE 4:**
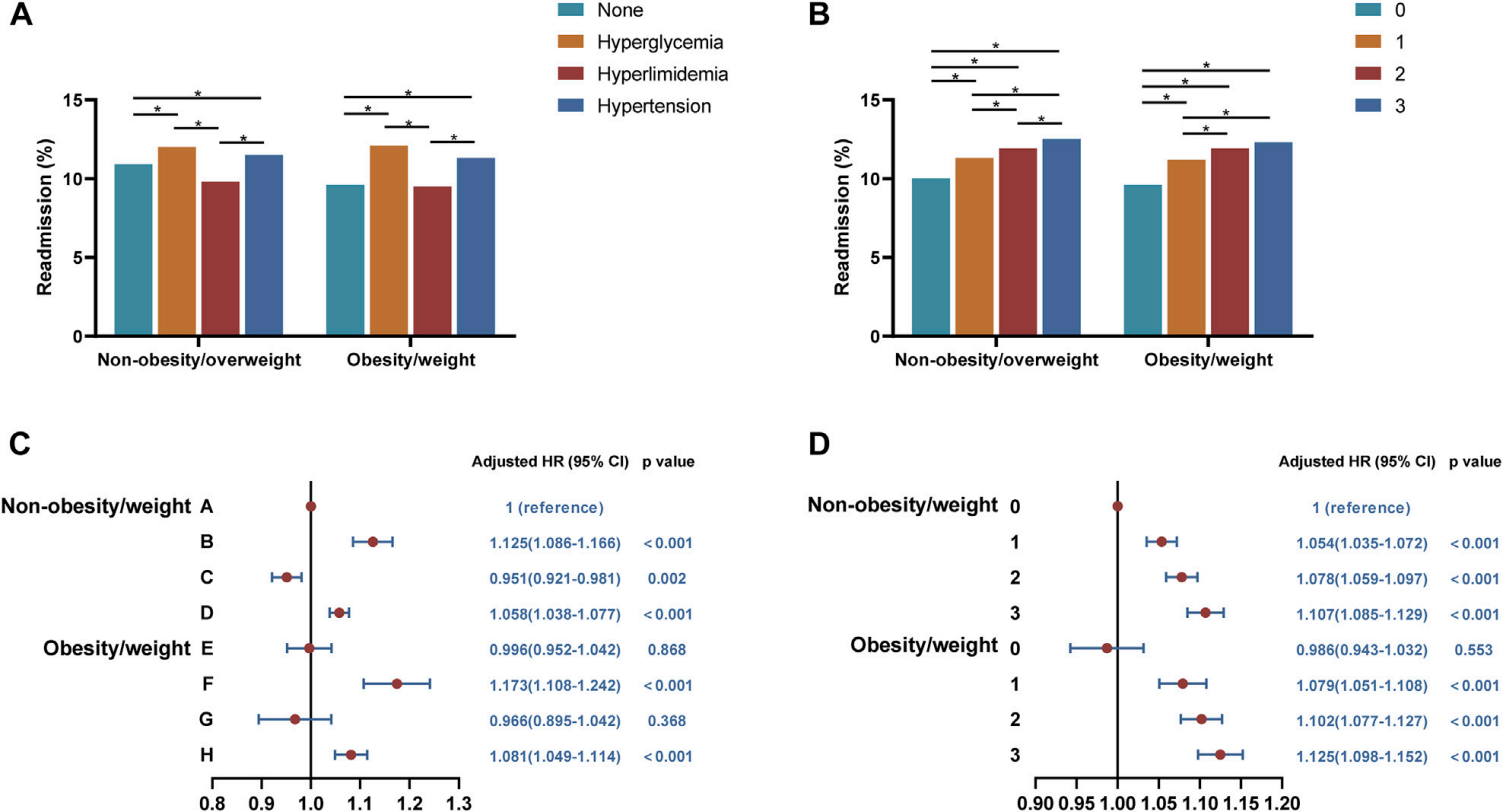
Analysis of the relationships between overweight/obesity with different types or amounts of metabolic abnormalities and 30-day readmissions. **(A)** Readmission rates in patients with overweight/obesity with different types of metabolic abnormalities. **(B)** Readmission rates in patients with overweight/obesity with different amounts of metabolic abnormalities. **(C)** Cox regression analysis of 30-day readmission in patients with overweight/obesity with different types of metabolic abnormalities. **(D)** Cox regression analysis of 30-day readmission in patients with overweight/obesity with different amounts of metabolic abnormalities. A, BMI < 25 kg/m^2^ and fewer than one metabolic syndrome component; B, BMI < 25 kg/m^2^ and only with hyperglycemia; C, BMI < 25 kg/m^2^ and only with hyperlipidemia; D, BMI < 25 kg/m^2^ and only with hypertension; E, BMI ≥ 25 kg/m^2^ and fewer than one metabolic syndrome component; F, BMI ≥ 25 kg/m^2^ and only with hyperglycemia; G, BMI ≥ 25 kg/m^2^ and only with hyperlipidemia; H, BMI ≥ 25 kg/m^2^ and only with hypertension.

Further analysis revealed that 30-day unplanned readmission rates were highest in patients only with hyperglycemia, followed by patients only with hypertension in both non-overweight/obesity and overweight/obesity groups (Figure 4A). According to Cox regression analysis, it could be concluded that the risks of 30-day unplanned readmission increased in patients with hyperglycemia (B group: HR, 1.125, 95%CI, 1.086–1.166, *p* < 0.001 and F group: HR, 1.173, 95%CI, 1.108–1.242, *p* < 0.001) or hypertension (D group: HR, 1.058, 95%CI, 1.108–1.242, *p* < 0.001 and H group: HR, 1.081, 95%CI,1.049–1.114, *p* < 0.001) (Figure 4C).

For overweight/obesity with different amounts of metabolic abnormalities, the 30-day unplanned readmission rate was significantly higher with two or three combined metabolic abnormalities than with only one metabolic abnormality and both were higher than without metabolic abnormalities regardless of overweight/obesity (Figure 4B). By Cox regression analysis, we found that the readmission risks of COPD patients with different amounts of metabolic abnormalities increased, and overweight/obesity alone did not affect the risks (Figure 4D).

## Discussion

In this nationally representative longitudinal study using NRD, we employed metabolic overweight/obesity phenotypes to distinguish metabolic status and overweight/obesity, making several key observations about the impact of metabolic overweight/obesity on hospitalized adults with COPD. We noted that the 30-day unplanned readmission risk increased in patients with MUNO and MUO compared to patients with MHNO. However, in patients with MHO, the risk did not significantly differ compared with MHNO. Interestingly, in elderly patients (≥65yr), MHO also exhibited a higher readmission risk, suggesting that the relationship between obesity and readmission risk may differ depending on age. Furthermore, regardless of overweight/obesity status, the readmission risk of COPD patients with hyperglycemia or hypertension increased.

Our study found that metabolic abnormalities had more serious effects on COPD patients than overweight/obesity. Consistent with our view, some studies suggested that the progression and prognosis of the disease can be aggravated as by the co-existence of MetS and COPD (Díez-Manglano et al., 2014; Chan et al., 2019). Patients with MetS and COPD faced a greater risk of cardiovascular comorbidity, a major cause of mortality (Sin and Man, 2003). The mechanisms behind these findings are related to systemic inflammation and oxidative stress which are crucial links connecting COPD to MetS (Chan et al., 2019). Both cross-sectional and longitudinal studies provide evidence that MetS in people with COPD worsens respiratory symptoms and lung function due to increased systemic inflammation (Stanciu et al., 2009; Cebron Lipovec et al., 2016; Price et al., 2016). The pro-inflammatory nature of oxidative stress is believed to be responsible for the increased cardiovascular comorbidity risk in COPD and MetS. Furthermore, individuals with COPD exhibit decreased levels of physical activity, decreasing the risk of various comorbidities. However, the presence of co-existing MetS further impairs physical activity in COPD patients (Clini et al., 2013). Moreover, MetS would reduce pulmonary function and accelerate the progression of COPD due to a lack of physical activity (Hartman et al., 2010).

In-depth investigations into the specific metabolic abnormalities that contribute to the burden of COPD patients and healthcare suggested that hyperglycemia and hypertension, but not hyperlipidemia, increased the risk of readmission regardless of overweight/obesity. Several studies supporting our conclusion indicated that COPD patients with hyperglycemia had a higher risk of death, long inpatient stay, and adverse acute exacerbations of COPD outcomes (Baker et al., 2006; Chakrabarti et al., 2009; Parappil et al., 2010). Various related mechanisms have been proposed: hyperglycemia-induced reactive oxygen species can impair lung function through activation of cellular stress pathways (Tiengo et al., 2008); hyperglycemia increases airway hyperresponsiveness through specific cellular pathways mediated by Rho-kinase (Cazzola et al., 2012); hyperglycemia also increases the risk of lung infections due to the presence of glucose in airway secretion, etc. (McKeever et al., 2005; Cazzola et al., 2012). As for COPD combined with hypertension, a secondary cohort study reported that COPD patients with cardiovascular disease (CVD) or CVD-related risk factors including hypertension are more likely to experience a subsequent CVD event following an exacerbation, especially when hospitalized within the first 30 days. This finding partly explains the increased risk of 30-day unplanned readmission in COPD patients with hypertension in our study. In addition, the more the number of combined metabolic abnormalities, the higher the readmission rate. If metabolic abnormality was regarded as comorbidity, consistent with our view, past studies indicated that the readmission risk would raise as the number of comorbidities increased in patients with COPD who combined with comorbidities (Roberts et al., 2011; Lin et al., 2020).

In addition, we explored other indicators of COPD burden including LOS, total charges, and in-hospital mortality within 30 days of readmission. Interestingly, the effect of metabolic obesity phenotypes on these indicators was inconsistent with their effect on 30-day unplanned readmission. Our study revealed that overweight/obesity had a favorable effect on in-hospital mortality for readmissions within 30 days. This result was consistent with several studies indicating the positive association between overweight/obesity and higher survival, supporting the “obesity paradox.”

This study, to our knowledge, is the first to report the relationship between metabolic overweight/obesity phenotypes and unplanned readmission in patients with COPD. The main strength of the study is that the NRD provided us a large nationally representative sample of patients, enabling the generalization of findings across various healthcare settings and socioeconomic backgrounds. In addition, our study considered basic characteristics and socioeconomic factors of patients and complications that reflect the severity of the patient’s disease. Besides, we also performed subgroup analyses according to sex, age, and with or without respiratory ventilation to minimize the effect of potential confounding factors on hospitalization outcomes among COPD patients with different metabolic overweight/obesity phenotypes. In spite of the strength of our study, there are some limitations. Firstly, the study relies on ICD10 and PCS codes for establishing diagnoses and inexact coding may lead to potential misclassification of exposure, outcomes, and covariates. Secondly, we lack data on death after discharge from hospital. However, we used readmissions within 30 days recommended by HCUP to minimize the bias. The third limitation is the absence of information on severity of COPD and complexity of index hospitalization which may confound the risk of 30-day unplanned readmission. However, we adjusted several factors including LOS, total charges, comorbidity score, etc. Lastly, in the NRD database, laboratory values, imaging, and medication data are not captured. Therefore, the severity of COPD patients’ condition and the definition of certain terms, such as metabolic syndrome, are not precise enough.

## Conclusion

In patients with COPD, overweight/obesity alone had little effect on unplanned readmission, whereas metabolic abnormalities, irrespective of overweight/obesity status, were associated with an increased risk of unplanned readmission. It suggested that abnormal metabolic status modified the effect of overweight/obesity on COPD. Concerning different metabolic abnormalities, special attention should be given to hyperglycemia and hypertension for patients with COPD. However, in elderly individuals (≥65yr) both overweight/obesity and metabolic abnormalities independently exacerbated the adverse outcomes. Therefore, effective clinical prevention and intervention strategies for COPD patients should prioritize the management of metabolic abnormalities, especially hyperglycemia and hypertension, in order to improve the clinical course and prognosis of COPD.

## Data Availability

The original contributions presented in the study are included in the article/Supplementary Material, further inquiries can be directed to the corresponding authors.

## Abbreviations

COPD, chronic obstructive pulmonary disease; BMI, body mass index; NRD, Nationwide Readmissions Database; MHNO, metabolically healthy non-overweight/obesity; MUNO, metabolically unhealthy non-overweight/obesity; MHO, metabolically healthy with overweight/obesity; MUO, metabolically unhealthy with overweight/obesity; HR, hazard ratio; CI, confidence interval; CMS, Centers for Medicare and Medicaid Services; HCUP, Healthcare Cost and Utilization Project; SID, State Inpatient Databases; ICD-10-CM, International Classification of Diseases, Tenth Revision, Clinical Modification; LOS, length of stay; PCS, Procedure Coding System; AIDS, Acquired Immune Deficiency Syndrome; MetS, metabolic syndrome; CVD, cardiovascular disease.
