# Supplementary material for: The impact of metabolic overweight/obesity phenotypes on unplanned readmission risk in patients with COPD: a retrospective cohort study
Source: Front Physiol. 2023 Nov 28;14:1290611. doi: 10.3389/fphys.2023.1290611 (PMC10714943; doi:10.3389/fphys.2023.1290611)
Supplement: Supplementary file 1 [file Table1.docx]

| **Supplementary Table 1 Diagnosis Codes, Used in Including and Excluding Patients** | | |
| --- | --- | --- |
| **Diagnoses** | | **ICD-10 Codes** |
| **COPD** | | C3410, C3411, C3412, C342, C3430, C3431, C3432, C3480, C3481, C3482, C3400, C3401, C3402, C3490, C3491, C3492 |
| **Pregnancy** | | Z331, Z332, Z333, Z3400, Z3401, Z3402, Z3403, Z3480, Z3481, Z3482, Z3483, Z3491, Z3492, Z3493, Z36, Z360, Z361, Z362, Z363, Z364, Z365, Z3681, Z3682, Z3683, Z3684, Z3685, Z3686, Z3687, Z3688, Z3689, Z368A, Z369, Z3A00, Z3A01, Z3A08, Z3A09, Z3A10, Z3A11, Z3A12, Z3A13, Z3A14, Z3A15, Z3A16, Z3A17, Z3A19, Z3A18, Z3A20, Z3A21, Z3A22, Z3A23, Z3A24, Z3A25, Z3A26, Z3A27, Z3A28, Z3A29, Z3A30, Z3A31, Z3A32, Z3A33, Z3A34, Z3A35, Z3A36, Z3A37, Z3A38, Z3A39, Z3A40, Z3A41, Z3A42, Z3A49, O000, O0000, O0001 |
| **Low body weight** | | Z681 |
| **Normal body weight** | | Z6820, Z6821, Z6822, Z6823, Z6824 |
| **Overweight** | | Z6825, Z6826, Z6827, Z6828, Z6829, E663, DE660A, E669O |
| **Obesity of grade 1** | | Z6830, Z6831, Z6832, Z6833, Z6834, DE660B |
| **Obesity of grade 2** | | Z6835, Z6836, Z6837, Z6838, Z6839, DE660C |
| **Obesity of grade 3** | | Z6841, Z6842, Z6843, Z6844, Z6845, E6601, E662, DE660E, DE660F, DE660G, DE660H |
| **Hyperglycemia** | \| **Prediabetes** \| \| --- \| \| R7301, R7302, R7303 \| \| **T1DM** \| \| E108, E109, E1010, E1011, E1021, E1022, E1029, E10311, E10319, E10321, E103213, E103212, E103211, E103219, E10329, E103293, E103292, E103291, E103299, E10331, E103313, E103312, E103311, E103319, E10339, E103393, E103392, E103391, E103399, E10341, E103413, E103412, E103411, E103419, E10349, E103493, E103492, E103491, E103499, E10351, E103513, E103512, E103511, E103519, E103523, E103522, E103521, E103529, E103533, E103532, E103531, E103539, E103543, E103542, E103541, E103549, E103553, E103552, E103551, E103559, E10359, E103593, E103592, E103591, E103599, E1036, E1039, E1037X3, E1037X2, E1037X1, E1037X9, E1040, E1041, E1042, E1044, E1043, E1049, E1051, E1052, E1059, E10610, E10618, E10620, E10621, E10622, E10628, E10630, E10638, E10641, E10649, E1065, E1069 \| \| **T2DM** \| \| E1100, E1101, E1111, E1110, E1122, E1121, E1129, E11311, E11319, E11321, E113213, E113212, E113211, E113219, E11329, E113293, E113292, E113291, E113299, E11331, E113313, E113312, E113311, E113319, E11339, E113393, E113392, E113391, E113399, E11341, E113413, E113412, E113411, E113419, E11349, E113493, E113492, E113491, E113499, E11351, E113513, E113512, E113511, E113519, E113523, E113522, E113521, E113529, E113533, E113532, E113531, E113539, E113543, E113542, E113541, E113549, E113553, E113552, E113551, E113559, E11359, E113593, E113592, E113591, E113599, E1136, E1139, E1144, E1143, E1141, E1140, E1142, E1149, E1152, E1151, E1159, E11620, E11621, E11622, E11628, E11610, E11618, E11630, E11638, E1137X3, E1137X2, E1137X1, E1137X9, E11641, E11649, E1165, E1169, E118, E119 \| \|  \| \| **Diabetic complication** \| \| E0800, E0801, E0810, E0811, E0821, E0829, E08311, E08319, E08321, E083211, E083212, E083213, E083219, E08329, E083291, E083292, E083293, E083299, E08331, E083311, E083312, E083313, E083319, E08339, E083391, E083392, E083393, E083399, E08341, E083411, E083412, E083413, E083419, E08349, E083491, E083492, E083493, E083499, E08351, E083511, E083512, E083513, E083519, E083521, E083522, E083523, E083529, E083531, E083532, E083543, E083549, E083551, E083552, E083553, E083559, E08359, E083591, E083592, E083593, E083599, E0836, E0837X1, E0837X2, E0837X3, E0837X9, E0839, E0840, E0841, E0842, E0843, E0844, E0849, E0851, E0852, E0859, E08610, E08618, E08620, E08621, E08622, E08628, E08630, E08638, E08641, E08649, E0865, E0869, E088, E089 \| \| **Others** \| \| E1300, E1301, E1310, E1311, E1321, E1322, E1329, E13311, E13319, E13321, E133213, E133212, E133211, E133219, E13329, E133293, E133292, E133291, E133299,E13331, E133313, E133312, E133311, E133319, E13339, E133393, E133392, E133391, E133399, E13341, E133413, E133412, E133411, E133419, E13349, E133493, E133492, E133491, E133499, E13351, E133513, E133512, E133511, E133519, E133523, E133522, E133521, E133529, E133533, E133532, E133531, E133539, E133543, E133542, E133541, E133549, E133553, E133552, E133551, E133559, E13359, E133593, E133592, E133591, E133599, E1340, E1341, E1342, E1343, E1344, E1349, E1336, E1339, E1351, E1352, E1359, E13610, E13618, E13620, E13621, E13622, E13630, E13638, E13641, E13649, E1365, E1369, E1337X3, E1337X2, E1337X1, E1337X9, E138, E139 \| | |
| **Hyperlipidemia** | \| **Hypercholesteremia** \| \| --- \| \| E780, E7800, E7801 \| \| **Hypertriglyceridemia** \| \| E781 \| \| **Others** \| \| E782, E784, E7849, E785, E783, E7841 \| | |
| **Hypertension** | | H35031, H35032, H35033, H35039, G932, I10, I110, I119, I120, I129, I130, I1310, I1311, I132, I150, I151, I152, I158, I159, I160, I161, I169, I674, R030, I973 |
| **Myocardial infarction** | | I2101, I2102, I2109, I2111, I2119, I2121, I2129, I213, I214, I219, I21A1, I21A9, I220, I221, I222, I228, I229, I252 |
| **Congestive heart failure** | | I501, I5020, I5021, I5022, I5023, I5030, I5031, I5032, I5033, I5040, I5041, I5042, I5043, I50810, I50811, I50812, I50813, I50814, I508, I5083, I5084, I5089, I509, I110,  I130, I132 |
| **Peripheral vascular disease** | | I700, I701, I70201, I70202, I70203, I70208, I70209, I70211, I70212, I70213, I70218, I70219, I70221, I70222, I70223, I70228, I70229, I70231, I70232, I70233, I70234, I70235, I70238, I70239, I70241, I70242, I70243, I70244, I70245, I70248, I70249, I7025, I70261, I70262, I70263, I70268, I70269, I70291, I70292, I70293, I70298, I70299, I70301, I70302, I70303, I70308, I70309, I70311, I70312, I70313, I70318, I70319, I70321, I70322, I70323, I70328, I70329, I70331, I70332, I70333, I70334, I70335, I70338, I70339, I70341, I70342, I70343, I70344 I70345, I70348, I70349, I7035, I70361, I70362, I70363, I70368, I70369, I70391, I70392, I70393, I70398, I70399, I70401, I70402, I70403, I70408, I70409, I70411, I70412, I70413, I70418, I70419, I70421, I70422, I70423, I70428, I70429, I70431, I70432, I70433, I70434, I70435, I70438, I70439, I70441, I70442, I70443, I70444, I70445, I70448, I70449, I7045, I70461, I70462, I70463, I70468, I70469, I70491, I70492, I70493, I70498, I70499, I70501, I70502, I70503, I70508, I70509, I70511, I70512, I70513, I70518, I70519, I70521, I70522, I70523, I70528, I70529, I70531, I70532, I70533, I70534, I70535, I70538, I70539, I70541, I70542, I70543, I70544, I70545, I70548, I70549, I7055, I70561, I70562, I70563, I70568, I70569, I70591, I70592, I70593, I70598, I70599, I70601, I70602, I70603, I70608, I70609, I70611, I70612, I70613, I70618, I70619, I70621, I70622, I70623, I70628, I70629, I70631, I70632, I70633, I70634, I70635, I70638, I70639, I70641, I70642, I70643, I70644, I70645, I70648, I70649, I7065, I70661, I70662, I70663, I70668, I70669, I70691, I70692, I70693, I70698, I70699, I70701, I70702, I70703, I70708, I70709, I70711, I70712, I70713, I70718, I70719, I70721, I70722, I70723, I70728, I70729, I70731, I70732, I70733, I70734, I70735, I70738, I70739, I70741, I70742, I70743, I70744, I70745, I70748, I70749, I7075, I70761, I70762, I70763, I70768, I70769, I70791, I70792, I70793, I70798, I70799, I708, I7090, I7091, I7092, I7100, I7101, I7102, I7103, I711, I712, I713, I714, I715, I716, I718, I719, I720, I721, I722, I723, I724, I725, I726, I728, I729, I7300, I7301, I731, I7381, I7389, I739, I7401, I7409, I7410, I7411, I7419, I742, I743, I744, I745, I748, I749, I770, I771, I772, I773, I774, I775, I776, I7770, I7771, I7772, I7773, I7774, I7775, I7776, I7777, I7779, I77810, I77811, I77812, I77819, I7789, I779 |
| **Cerebrovascular disease** | | I6000, I6001, I6002, I6010, I6011, I6012, I602, I6020, I6021, I6022, I6030, I6031, I6032, I604, I6050, I6051, I6052, I606, I607, I608, I609, I610, I611, I612, I613, I614, I615, I616, I618, I619, I6200, I6201, I6202, I6203, I621, I629, I6300, I63011, I63012, I63013, I63019, I6302, I63031, I63032, I63033, I63039, I6309, I6310, I63111, I63112, I63113, I63119, I6312, I63131, I63132, I63133, I63139, I6319, I6320, I63211, I63212, I63213, I63219, I6322, I63231, I63232, I63233, I63239, I6329, I6330, I63311, I63312, I63313, I63319, I63321, I63322, I63323, I63329, I63331, I63332, I63333, I63339, I63341, I63342, I63343, I63349, I6339, I6340, I63411, I63412, I63413, I63419, I63421, I63422, I63423, I63429, I63431, I63432, I63433, I63439, I63441, I63442, I63443, I63449, I6349, I6350, I63511, I63512, I63513, I63519, I63521, I63522, I63523, I63529, I63531, I63532, I63533, I63539, I63541, I63542, I63543, I63549, I6359, I636, I638, I6381, I6389, I639, I6501, I6502, I6503, I6509, I651, I6521, I6522, I6523, I6529, I658, I659, I6601, I6602, I6603, I6609, I6611, I6612, I6613, I6619, I6621, I6622, I6623, I6629, I663, I668, I669, I670, I671, I672, I673, I674, I675, I676, I677, I6781, I6782, I6783, I67841, I67848, I67850, I67858, I6789, I679, I680, I682, I688, I6900, I6901, I69010, I69011, I69012, I69013, I69014, I69015, I69018, I69019, I69020, I69021, I69022, I69023, I69028, I69031, I69032, I69033, I69034, I69039, I69041, I69042, I69043, I69044, I69049, I69051, I69052, I69053, I69054, I69059, I69061, I69062, I69063, I69064, I69065, I69069, I69090, I69091, I69092, I69093, I69098, I6910, I6911, I69110, I69111, I69112, I69113, I69114, I69115, I69118, I69119, I69120, I69121, I69122, I69123, I69128, I69131, I69132, I69133, I69134, I69139, I69141, I69142, I69143, I69144, I69149, I69151, I69152, I69153, I69154, I69159, I69161, I69162, I69163, I69164, I69165, I69169, I69190, I69191, I69192, I69193, I69198, I6920, I6921, I69210, I69211, I69212, I69213, I69214, I69215, I69218, I69219, I69220, I69221, I69222, I69223, I69228, I69231, I69232, I69233, I69234, I69239, I69241, I69242, I69243, I69244, I69249, I69251, I69252, I69253, I69254, I69259, I69261, I69262, I69263, I69264, I69265, I69269, I69290, I69291, I69292, I69293, I69298, I6930, I6931, I69310, I69311, I69312, I69313, I69314, I69315, I69318, I69319, I69320, I69321, I69322, I69323, I69328, I69331, I69332, I69333, I69334, I69339, I69341, I69342, I69343, I69344, I69349, I69351, I69352, I69353, I69354, I69359, I69361, I69362, I69363, I69364, I69365, I69369, I69390, I69391, I69392, I69393, I69398, I6980, I6981, I69810, I69811, I69812, I69813, I69814, I69815, I69818, I69819, I69820, I69821, I69822, I69823, I69828, I69831, I69832, I69833, I69834, I69839, I69841, I69842, I69843, I69844, I69849, I69851, I69852, I69853, I69854, I69859, I69861, I69862, I69863, I69864, I69865, I69869, I69890, I69891, I69892, I69893, I69898, I6990, I6991, I69910, I69911, I69912, I69913, I69914, I69915, I69918, I69919, I69920, I69921, I69922, I69923, I69928, I69931, I69932, I69933, I69934, I69939, I69941, I69942, I69943, I69944, I69949, I69951, I69952, I69953, I69954, I69959, I69961, I69962, I69963, I69964, I69965, I69969, I69990, I69991, I69992, I69993, I69998, G450, G451, G452, G453, G454, G458, G459, G460, G461, G462, G463, G464, G465, G466, G467, G468 |
| **Dementia** | | F0150, F0151, F0280, F0281, F0390, F0391, G300, G301, G308, G309, G3101, G3109, G311, G312, G3181, G3182, G3183, G3184, G3185, G3189, G319 |
| **Connective tissue disease** | | M0500, M05011, M05012, M05019, M05021, M05022, M05029, M05031, M05032, M05039, M05041, M05042, M05049, M05051, M05052, M05059, M05061, M05062, M05069, M05071, M05072, M05079, M0509, M0510, M05111, M05112, M05119, M05121, M05122, M05129, M05131, M05132, M05139, M05141, M05142, M05149, M05151, M05152, M05159, M05161, M05162, M05169, M05171, M05172, M05179, M0519, M0520, M05211, M05212, M05219, M05221, M05222, M05229, M05231, M05232, M05239, M05241, M05242, M05249, M05251, M05252, M05259, M05261, M05262, M05269, M05271, M05272, M05279, M0529, M0530, M05311, M05312, M05319, M05321, M05322, M05329, M05331, M05332, M05339, M05341, M05342, M05349, M05351, M05352, M05359, M05361, M05362, M05369, M05371, M05372, M05379, M0539, M0540, M05411, M05412, M05419, M05421, M05422, M05429, M05431, M05432, M05439, M05441, M05442, M05449, M05451, M05452, M05459, M05461, M05462, M05469, M05471, M05472, M05479, M0549, M0550, M05511, M05512, M05519, M05521, M05522, M05529, M05531, M05532, M05539, M05541, M05542, M05549, M05551, M05552, M05559, M05561, M05562, M05569, M05571, M05572, M05579, M0559, M0560, M05611, M05612, M05619, M05621, M05622, M05629, M05631, M05632, M05639, M05641, M05642, M05649, M05651, M05652, M05659, M05661, M05662, M05669, M05671, M05672, M05679, M0569, M0570, M05711, M05712, M05719, M05721, M05722, M05729, M05731, M05732, M05739, M05741, M05742, M05749, M05751, M05752, M05759, M05761, M05762, M05769, M05771, M05772, M05779, M0579, M057A, M0580, M05811, M05812, M05819, M05821, M05822, M05829, M05831, M05832, M05839, M05841, M05842, M05849, M05851, M05852, M05859, M05861, M05862, M05869, M05871, M05872, M05879, M0589, M058A, M059, M0600, M06011, M06012, M06019, M06021, M06022, M06029, M06031, M06032, M06039, M06041, M06042, M06049, M06051, M06052, M06059, M06061, M06062, M06069, M06071, M06072, M06079, M0608, M0609, M060A, M061, M0620, M06211, M06212, M06219, M06221, M06222, M06229, M06231, M06232, M06239, M06241, M06242, M06249, M06251, M06252, M06259, M06261, M06262, M06269, M06271, M06272, M06279, M0628, M0629, M0630, M06311, M06312, M06319, M06321, M06322, M06329, M06331, M06332, M06339, M06341, M06342, M06349, M06351, M06352, M06359, M06361, M06362, M06369, M06371, M06372, M06379, M0638, M0639, M064, M0680, M06811, M06812, M06819, M06821, M06822, M06829, M06831, M06832, M06839, M06841, M06842, M06849, M06851, M06852, M06859, M06861, M06862, M06869, M06871, M06872, M06879, M0688, M0689, M068A, M069, M0800, M08011, M08012, M08019, M08021, M08022, M08029, M08031, M08032, M08039, M08041, M08042, M08049, M08051, M08052, M08059, M08061, M08062, M08069, M08071, M08072, M08079, M0808, M0809, M080A, M081, M0820, M08211, M08212, M08219, M08221, M08222, M08229, M08231, M08232, M08239, M08241, M08242, M08249, M08251, M08252, M08259, M08261, M08262, M08269, M08271, M08272, M08279, M0828, M0829, M082A, M083, M0840, M08411, M08412, M08419, M08421, M08422, M08429, M08431, M08432, M08439, M08441, M08442, M08449, M08451, M08452, M08459, M08461, M08462, M08469, M08471, M08472, M08479, M0848, M084A, M0880, M08811, M08812, M08819, M08821, M08822, M08829, M08831, M08832, M08839, M08841, M08842, M08849, M08851, M08852, M08859, M08861, M08862, M08869, M08871, M08872, M08879, M0888, M0889, M0890, M08911, M08912, M08919, M08921, M08922, M08929, M08931, M08932, M08939, M08941, M08942, M08949, M08951, M08952, M08959, M08961, M08962, M08969, M08971, M08972, M08979, M0898, M0899, M089A, M300, M301, M302, M303, M308, M310, M311, M312, M3130, M3131, M314, M315, M316, M317, M318, M319, M320, M3210, M3211, M3212, M3213, M3214, M3215, M3219, M328, M329, M3300, M3301, M3302, M3303, M3309, M3310, M3311, M3312, M3313, M3319, M3320, M3321, M3322, M3329, M3390, M3391, M3392, M3393, M3399, M340, M341, M342, M3481, M3482, M3483, M3489, M349, M3500, M3501, M3502, M3503, M3504, M3509, M351, M352, M353, M354, M355, M356, M357, M358, M3581, M3589, M359, M360, M361, M362, M363, M364, M368 |
| **Ulcer disease** | | K250, K251, K252, K253, K254, K255, K256, K257, K259, K260, K261, K262, K263, K264, K265, K266, K267, K269, K270, K271, K272, K273, K274, K275, K276,K277, K279, K280, K281, K282, K283, K284, K285, K286, K287, K289, K2210, K2211 |
| **Mild liver disease** | | B150, B159, B160, B161, B162, B169, B170, B1710, B1711, B172, B178, B179, B180, B181, B182, B188, B189, B190, B1910, B1911, B1920, B1921, B199, K700, K7010, K7011, K702, K7030, K7031, K709, K710, K7110, K7111, K712, K713, K714, K7150, K7151, K716, K717, K718, K719, K730, K731, K732, K738, K739, K740, K7400, K7401, K7402, K741, K742, K743, K744, K745, K7460, K7469, K760, K761, K762, K763, K764, K765, K766, K767, K7681, K7689, K769 |
| **Hemiplegia** | | G800, G801, G802, G803, G804, G808, G809, G8100, G8101, G8102, G8103, G8104, G8110, G8111, G8112, G8113, G8114, G8190, G8191, G8192, G8193, G8194, G8220, G8221, G8222, G8250, G8251, G8252, G8253, G8254, G830, G8310, G8311, G8312, G8313, G8314, G8320, G8321, G8322, G8323, G8324, G8330, G8331, G8332, G8333, G8334, G834, G835, G8381, G8382, G8383, G8384, G8389, G839 |
| **Moderate or severe renal disease** | | I120, I129, I130, I1310, I1311, I132, N000, N001, N002, N003, N004, N005, N006, N007, N008, N009, N00A, N010, N011, N012, N013, N014, N015, N016, N017, N018, N019, N01A, N020, N021, N022, N023, N024, N025, N026, N027, N028, N029, N02A, N030, N031, N032, N033, N034, N035, N036, N037, N038, N039, N03A, N040, N041, N042, N043, N044, N045, N046, N047, N048, N049, N04A, N050, N051, N052, N053, N054, N055, N056, N057, N058, N059, N05A, N070, N071, N072, N073, N074, N075, N076, N077, N078, N079, N07A, N110, N111, N118, N119, N140, N141, N142, N143, N144, N170, N171, N172, N178, N179, N181, N182, N183, N1830, N1831, N1832, N184, N185, N186, N189, N19 |
| **Any tumor** | | C000, C001, C002, C003, C004, C005, C006, C008, C009, C01, C020, C021, C022, C023, C024, C028, C029, C030, C031, C039, C040, C041, C048, C049, C050, C051, C052, C058, C059, C060, C061, C062, C0680, C0689, C069, C07, C080, C081, C089, C090, C091, C098, C099, C100, C101, C102, C103, C104, C108, C109, C110, C111, C112, C113, C118, C119, C12, C130, C131, C132, C138, C139, C140, C142, C148, C153, C154, C155, C158, C159, C160, C161, C162, C163, C164, C165, C166, C168, C169, C170, C171, C172, C173, C178, C179, C180, C181, C182, C183, C184, C185, C186, C187, C188, C189, C19, C20, C210, C211, C212, C218, C220, C221, C222, C223, C224, C227, C228, C229, C23, C240, C241, C248, C249, C250, C251, C252, C253, C254, C257, C258, C259, C260, C261, C269, C300, C301, C310, C311, C312, C313, C318, C319, C320, C321, C322, C323, C328, C329, C33, C3400, C3401, C3402, C3410, C3411, C3412, C342, C3430, C3431, C3432, C3480, C3481, C3482, C3490, C3491, C3492, C37, C380, C381, C382, C383, C384, C388, C390, C399, C4000, C4001, C4002, C4010, C4011, C4012, C4020, C4021, C4022, C4030, C4031, C4032, C4080, C4081, C4082, C4090, C4091, C4092, C410, C411, C412, C413, C414, C419, C430, C4310, C4311, C43111, C43112, C4312, C43121, C43122, C4320, C4321, C4322, C4330, C4331, C4339, C434, C4351, C4352, C4359, C4360, C4361, C4362, C4370, C4371, C4372, C438, C439, C4400, C4401, C4402, C4409, C44101, C44102, C441021, C441022, C44109, C441091, C441092, C44111, C44112, C441121, C441122, C44119, C441191, C441192, C44121, C44122, C441221, C441222, C44129, C441291, C441292, C44131, C441321, C441322, C441391, C441392, C44191, C44192, C441921, C441922, C44199, C441991, C441992, C44201, C44202, C44209, C44211, C44212, C44219, C44221, C44222, C44229, C44291, C44292, C44299, C44300, C44301, C44309, C44310, C44311, C44319, C44320, C44321, C44329, C44390, C44391, C44399, C4440, C4441, C4442, C4449, C44500, C44501, C44509, C44510, C44511, C44519, C44520, C44521, C44529, C44590, C44591, C44599, C44601, C44602, C44609, C44611, C44612, C44619, C44621, C44622, C44629, C44691, C44692, C44699, C44701, C44702, C44709, C44711, C44712, C44719, C44721, C44722, C44729, C44791, C44792, C44799, C4480, C4481, C4482, C4489, C4490, C4491, C4492, C4499, C450, C451, C452, C457, C459, C460, C461, C462, C463, C464, C4650, C4651, C4652, C467, C469, C470, C4710, C4711, C4712, C4720, C4721, C4722, C473, C474, C475, C476, C478, C479, C480, C481, C482, C488, C490, C4910, C4911, C4912, C4920, C4921, C4922, C493, C494, C495, C496, C498, C499, C49A0, C49A1, C49A2, C49A3, C49A4, C49A5, C49A9, C4A0, C4A10, C4A11, C4A111, C4A112, C4A12, C4A121, C4A122, C4A20, C4A21, C4A22, C4A30, C4A31, C4A39, C4A4, C4A51, C4A52, C4A59, C4A60, C4A61, C4A62, C4A70, C4A71, C4A72, C4A8, C4A9, C50011, C50012, C50019, C50021, C50022, C50029, C50111, C50112, C50119, C50121, C50122, C50129, C50211, C50212, C50219, C50221, C50222, C50229, C50311, C50312, C50319, C50321, C50322, C50329, C50411, C50412, C50419, C50421, C50422, C50429, C50511, C50512, C50519, C50521, C50522, C50529, C50611, C50612, C50619, C50621, C50622, C50629, C50811, C50812, C50819, C50821, C50822, C50829, C50911, C50912, C50919, C50921, C50922, C50929, C510, C511, C512, C518, C519, C52, C530, C531, C538, C539, C540, C541, C542, C543, C548, C549, C55, C561, C562, C569, C5700, C5701, C5702, C5710, C5711, C5712, C5720, C5721, C5722, C573, C574, C577, C578, C579, C58, C600, C601, C602, C608, C609, C61, C6200, C6201, C6202, C6210, C6211, C6212, C6290, C6291, C6292, C6300, C6301, C6302, C6310, C6311, C6312, C632, C637, C638, C639, C641, C642, C649, C651, C652, C659, C661, C662, C669, C670, C671, C672, C673, C674, C675, C676, C677, C678, C679, C680, C681, C688, C689, C6900, C6901, C6902, C6910, C6911, C6912, C6920, C6921, C6922, C6930, C6931, C6932, C6940, C6941, C6942, C6950, C6951, C6952, C6960, C6961, C6962, C6980, C6981, C6982, C6990, C6991, C6992, C700, C701, C709, C710, C711, C712, C713, C714, C715, C716, C717, C718, C719, C720, C721, C7220, C7221, C7222, C7230, C7231, C7232, C7240, C7241, C7242, C7250, C7259, C729, C73, C7400, C7401, C7402, C7410, C7411, C7412, C7490, C7491, C7492, C750, C751, C752, C753, C754, C755, C758, C759, C760, C761, C762, C763, C7640, C7641, C7642, C7650, C7651, C7652, C768, C770, C771, C772, C773, C774, C775, C778, C779, C7800, C7801, C7802, C781, C782, C7830, C7839, C784, C785, C786, C787, C7880, C7889, C7900, C7901, C7902, C7910, C7911, C7919, C792, C7931, C7932, C7940, C7949, C7951, C7952, C7960, C7961, C7962, C7970, C7971, C7972, C7981, C7982, C7989, C799, C7A00, C7A010, C7A011, C7A012, C7A019, C7A020, C7A021, C7A022, C7A023, C7A024, C7A025, C7A026, C7A029, C7A090, C7A091, C7A092, C7A093, C7A094, C7A095, C7A096, C7A098, C7A1, C7A8, C7B00, C7B01, C7B02, C7B03, C7B04, C7B09, C7B1, C7B8, C800, C801, C802, C8100, C8101, C8102, C8103, C8104, C8105, C8106, C8107, C8108, C8109, C8110, C8111, C8112, C8113, C8114, C8115, C8116, C8117, C8118, C8119, C8120, C8121, C8122, C8123, C8124, C8125, C8126, C8127, C8128, C8129, C8130, C8131, C8132, C8133, C8134, C8135, C8136, C8137, C8138, C8139, C8140, C8141, C8142, C8143, C8144, C8145, C8146, C8147, C8148, C8149, C8170, C8171, C8172, C8173, C8174, C8175, C8176, C8177, C8178, C8179, C8190, C8191, C8192, C8193, C8194, C8195, C8196, C8197, C8198, C8199, C8200, C8201, C8202, C8203, C8204, C8205, C8206, C8207, C8208, C8209, C8210, C8211, C8212, C8213, C8214, C8215, C8216, C8217, C8218, C8219, C8220, C8221, C8222, C8223, C8224, C8225, C8226, C8227, C8228, C8229, C8230, C8231, C8232, C8233, C8234, C8235, C8236, C8237, C8238, C8239, C8240, C8241, C8242, C8243, C8244, C8245, C8246, C8247, C8248, C8249, C8250, C8251, C8252, C8253, C8254, C8255, C8256, C8257, C8258, C8259, C8260, C8261, C8262, C8263, C8264, C8265, C8266, C8267, C8268, C8269, C8280, C8281, C8282, C8283, C8284, C8285, C8286, C8287, C8288, C8289, C8290, C8291, C8292, C8293, C8294, C8295, C8296, C8297, C8298, C8299, C8300, C8301, C8302, C8303, C8304, C8305, C8306, C8307, C8308, C8309, C8310, C8311, C8312, C8313, C8314, C8315, C8316, C8317, C8318, C8319, C8330, C8331, C8332, C8333, C8334, C8335, C8336, C8337, C8338, C8339, C8350, C8351, C8352, C8353, C8354, C8355, C8356, C8357, C8358, C8359, C8370, C8371, C8372, C8373, C8374, C8375, C8376, C8377, C8378, C8379, C8380, C8381, C8382, C8383, C8384, C8385, C8386, C8387, C8388, C8389, C8390, C8391, C8392, C8393, C8394, C8395, C8396, C8397, C8398, C8399, C8400, C8401, C8402, C8403, C8404, C8405, C8406, C8407, C8408, C8409, C8410, C8411, C8412, C8413, C8414, C8415, C8416, C8417, C8418, C8419, C8440, C8441, C8442, C8443, C8444, C8445, C8446, C8447, C8448, C8449, C8460, C8461, C8462, C8463, C8464, C8465, C8466, C8467, C8468, C8469, C8470, C8471, C8472, C8473, C8474, C8475, C8476, C8477, C8478, C8479, C8490, C8491, C8492, C8493, C8494, C8495, C8496, C8497, C8498, C8499, C84A0, C84A1, C84A2, C84A3, C84A4, C84A5, C84A6, C84A7, C84A8, C84A9, C84Z0, C84Z1, C84Z2, C84Z3, C84Z4, C84Z5, C84Z6, C84Z7, C84Z8, C84Z9, C8510, C8511, C8512, C8513, C8514, C8515, C8516, C8517, C8518, C8519, C8520, C8521, C8522, C8523, C8524, C8525, C8526, C8527, C8528, C8529, C8580, C8581, C8582, C8583, C8584, C8585, C8586, C8587, C8588, C8589, C8590, C8591, C8592, C8593, C8594, C8595, C8596, C8597, C8598, C8599, C860, C861, C862, C863, C864, C865, C866, C880, C882, C883, C884, C888, C889, C9000, C9001, C9002, C9010, C9011, C9012, C9020, C9021, C9022, C9030, C9031, C9032, C9100, C9101, C9102, C9110, C9111, C9112, C9130, C9131, C9132, C9140, C9141, C9142, C9150, C9151, C9152, C9160, C9161, C9162, C9190, C9191, C9192, C91A0, C91A1, C91A2, C91Z0, C91Z1, C91Z2, C9200, C9201, C9202, C9210, C9211, C9212, C9220, C9221, C9222, C9230, C9231, C9232, C9240, C9241, C9242, C9250, C9251, C9252, C9260, C9261, C9262, C9290, C9291, C9292, C92A0, C92A1, C92A2, C92Z0, C92Z1, C92Z2, C9300, C9301, C9302, C9310, C9311, C9312, C9330, C9331, C9332, C9390, C9391, C9392, C93Z0, C93Z1, C93Z2, C9400, C9401, C9402, C9420, C9421, C9422, C9430, C9431, C9432, C9440, C9441, C9442, C946, C9480, C9481, C9482, C9500, C9501, C9502, C9510, C9511, C9512, C9590, C9591, C9592, C960, C962, C9620, C9621, C9622, C9629, C964, C965, C966, C969, C96A, C96Z, D0000, D0001, D0002, D0003, D0004, D0005, D0006, D0007, D0008, D001, D002, D010, D011, D012, D013, D0140, D0149, D015, D017, D019, D020, D021, D0220, D0221, D0222, D023, D024, D030, D0310, D0311, D03111, D03112, D0312, D03121, D03122, D0320, D0321, D0322, D0330, D0339, D034, D0351, D0352, D0359, D0360, D0361, D0362, D0370, D0371, D0372, D038, D039, D040, D0410, D0411, D04111, D04112, D0412, D04121, D04122, D0420, D0421, D0422, D0430, D0439, D044, D045, D0460, D0461, D0462, D0470, D0471, D0472, D048, D049, D0500, D0501, D0502, D0510, D0511, D0512, D0580, D0581, D0582, D0590, D0591, D0592, D060, D061, D067, D069, D070, D071, D072, D0730, D0739, D074, D075, D0760, D0761, D0769, D090, D0910, D0919, D0920, D0921, D0922, D093, D098, D099, D100, D101, D102, D1030, D1039, D104, D105, D106, D107, D109, D110, D117, D119, D120, D121, D122, D123, D124, D125, D126, D127, D128, D129, D130, D131, D132, D1330, D1339, D134, D135, D136, D137, D139, D140, D141, D142, D1430, D1431, D1432, D144, D150, D151, D152, D157, D159, D1600, D1601, D1602, D1610, D1611, D1612, D1620, D1621, D1622, D1630, D1631, D1632, D164, D165, D166, D167, D168, D169, D170, D171, D1720, D1721, D1722, D1723, D1724, D1730, D1739, D174, D175, D176, D1771, D1772, D1779, D179, D1800, D1801, D1802, D1803, D1809, D181, D190, D191, D197, D199, D200, D201, D210, D2110, D2111, D2112, D2120, D2121, D2122, D213, D214, D215, D216, D219, D220, D2210, D2211, D22111, D22112, D2212, D22121, D22122, D2220, D2221, D2222, D2230, D2239, D224, D225, D2260, D2261, D2262, D2270, D2271, D2272, D229, D230, D2310, D2311, D23111, D23112, D2312, D23121, D23122, D2320, D2321, D2322, D2330, D2339, D234, D235, D2360, D2361, D2362, D2370, D2371, D2372, D239, D241, D242, D249, D250, D251, D252, D259, D260, D261, D267, D269, D270, D271, D279, D280, D281, D282, D287, D289, D290, D291, D2920, D2921, D2922, D2930, D2931, D2932, D294, D298, D299, D3000, D3001, D3002, D3010, D3011, D3012, D3020, D3021, D3022, D303, D304, D308, D309, D3100, D3101, D3102, D3110, D3111, D3112, D3120, D3121, D3122, D3130, D3131, D3132, D3140, D3141, D3142, D3150, D3151, D3152, D3160, D3161, D3162, D3190, D3191, D3192, D320, D321, D329, D330, D331, D332, D333, D334, D337, D339, D34, D3500, D3501, D3502, D351, D352, D353, D354, D355, D356, D357, D359, D360, D3610, D3611, D3612, D3613, D3614, D3615, D3616, D3617, D367, D369, D3701, D3702, D37030, D37031, D37032, D37039, D3704, D3705, D3709, D371, D372, D373, D374, D375, D376, D378, D379, D380, D381, D382, D383, D384, D385, D386, D390, D3910, D3911, D3912, D392, D398, D399, D3A00, D3A010, D3A011, D3A012, D3A019, D3A020, D3A021, D3A022, D3A023, D3A024, D3A025, D3A026, D3A029, D3A090, D3A091, D3A092, D3A093, D3A094, D3A095, D3A096, D3A098, D3A8, D400, D4010, D4011, D4012, D408, D409, D4100, D4101, D4102, D4110, D4111, D4112, D4120, D4121, D4122, D413, D414, D418, D419, D420, D421, D429, D430, D431, D432, D433, D434, D438, D439, D440, D4410, D4411, D4412, D442, D443, D444, D445, D446, D447, D449, |
| **Leukemia** | | C9100, C9101, C9102, C9110, C9111, C9112, C9130, C9131, C9132, C9140, C9141, C9142, C9150, C9151, C9152, C9160, C9161, C9162, C9190, C9191, C9192, C91A0, C91A1, C91A2, C91Z0, C91Z1, C91Z2, C9200, C9201, C9202, C9210, C9211, C9212, C9220, C9221, C9222, C9230, C9231, C9232, C9240, C9241, C9242, C9250, C9251, C9252, C9260, C9261, C9262, C9290, C9291, C9292, C92A0, C92A1, C92A2, C92Z0, C92Z1, C92Z2, C9300, C9301, C9302, C9310, C9311, C9312, C9330, C9331, C9332, C9390, C9391, C9392, C93Z0, C93Z1, C93Z2, C9400, C9401, C9402, C9420, C9421, C9422, C9430, C9431, C9432, C9440, C9441, C9442, C946, C9480, C9481, C9482, C9500, C9501, C9502, C9510, C9511, C9512, C9590, C9591, C9592 |
| **Lymphoma** | | C960, C962, C9620, C9621, C9622, C9629, C964, C965, C966, C969, C96A, C96Z, C8100, C8101, C8102, C8103, C8104, C8105, C8106, C8107, C8108, C8109, C8110,C8111, C8112, C8113, C8114, C8115, C8116, C8117, C8118, C8119, C8120, C8121, C8122, C8123, C8124, C8125, C8126, C8127, C8128, C8129, C8130, C8131, C8132, C8133, C8134, C8135, C8136, C8137, C8138, C8139, C8140, C8141, C8142, C8143, C8144, C8145, C8146, C8147, C8148, C8149, C8170, C8171, C8172, C8173, C8174, C8175, C8176, C8177, C8178, C8179, C8190, C8191, C8192, C8193, C8194, C8195, C8196, C8197, C8198, C8199, C8200, C8201, C8202, C8203, C8204, C8205, C8206, C8207, C8208, C8209, C8210, C8211, C8212, C8213, C8214, C8215, C8216, C8217, C8218, C8219, C8220, C8221, C8222, C8223, C8224, C8225, C8226, C8227, C8228, C8229, C8230, C8231, C8232, C8233, C8234, C8235, C8236, C8237, C8238, C8239, C8240, C8241, C8242, C8243, C8244, C8245, C8246, C8247, C8248, C8249, C8250, C8251, C8252, C8253, C8254, C8255, C8256, C8257, C8258, C8259, C8260, C8261, C8262, C8263, C8264, C8265, C8266, C8267, C8268, C8269, C8280, C8281, C8282, C8283, C8284, C8285, C8286, C8287, C8288, C8289, C8290, C8291, C8292, C8293, C8294, C8295, C8296, C8297, C8298, C8299, C8300, C8301, C8302, C8303, C8304, C8305, C8306, C8307, C8308, C8309, C8310, C8311, C8312, C8313, C8314, C8315, C8316, C8317, C8318, C8319, C8330, C8331, C8332, C8333, C8334, C8335, C8336, C8337, C8338, C8339, C8350, C8351, C8352, C8353, C8354, C8355, C8356, C8357, C8358, C8359, C8370, C8371, C8372, C8373, C8374, C8375, C8376, C8377, C8378,  C8379, C8380, C8381, C8382, C8383, C8384, C8385, C8386, C8387, C8388, C8389, C8390, C8391, C8392, C8393, C8394, C8395, C8396, C8397, C8398, C8399, C8400, C8401, C8402, C8403, C8404, C8405, C8406, C8407, C8408, C8409, C8410, C8411, C8412, C8413, C8414, C8415, C8416, C8417, C8418, C8419, C8440, C8441, C8442, C8443, C8444, C8445, C8446, C8447, C8448, C8449, C8460, C8461, C8462, C8463, C8464, C8465, C8466, C8467, C8468, C8469, C8470, C8471, C8472, C8473, C8474, C8475, C8476, C8477, C8478, C8479, C8490, C8491, C8492, C8493, C8494, C8495, C8496, C8497, C8498, C8499, C84A0, C84A1, C84A2, C84A3, C84A4, C84A5, C84A6, C84A7, C84A8, C84A9, C84Z0, C84Z1, C84Z2, C84Z3, C84Z4, C84Z5, C84Z6, C84Z7, C84Z8, C84Z9, C8510, C8511, C8512, C8513, C8514, C8515, C8516, C8517, C8518, C8519, C8520, C8521, C8522, C8523, C8524, C8525, C8526, C8527, C8528, C8529, C8580, C8581, C8582, C8583, C8584, C8585, C8586, C8587, C8588, C8589, C8590, C8591, C8592, C8593, C8594, C8595, C8596, C8597, C8598, C8599, C860, C861, C862, C863, C864, C865, C866, C880, C882, C883, C884, C888, C889 |
| **Moderate/severe liver disease** | | K7040, K7041, K7200, K7201, K7210, K7211, K7290, K7291 |
| **AIDS** | | B20 |
| **Procedure** | | **ICD-10 PCS** |
| **Respiratory intubation and mechanical ventilation** | | 09HN7BZ, 09HN8BZ, 0BH13EZ, 0BH17EZ, 0BH18EZ, 0CHY7BZ, 0CHY8BZ, 0DH57BZ, 0DH58BZ, 0WHQ73Z, 0WHQ7YZ, 5A09357, 5A09457, 5A09557, 5A1935Z, 5A1945Z, 5A1955Z, 5A0920Z, 5A09358, 5A09359, 5A0935B, 5A0935Z, 5A09458, 5A09459, 5A0945B, 5A0945Z, 5A09558, 5A09559, 5A0955B, 5A0955Z, 5A19054 |

ICD, International Classification of Diseases.
